# The role of patient-related factors in the implementation of a multimodal home-based rehabilitation intervention after discharge from inpatient geriatric rehabilitation (GeRas): a qualitative process evaluation

**DOI:** 10.1007/s41999-024-01027-5

**Published:** 2024-07-31

**Authors:** Leonie Maier, Petra Benzinger, Bastian Abel, Patrick Roigk, Martin Bongartz, Isabel Wirth, Ingeborg Cuvelier, Sabine Schölch, Gisela Büchele, Oliver Deuster, Jürgen Bauer, Kilian Rapp, Charlotte Ullrich, Michel Wensing, Catharina Roth

**Affiliations:** 1 Department of General Practice and Health Services Research, Heidelberg University Hospital, Heidelberg, Germany; 2 Department of Clinical Gerontology, Robert-Bosch-Hospital, Stuttgart, Germany; 3 Center for Geriatric Medicine, Heidelberg University Hospital, AGAPLESION Bethanien Hospital Heidelberg, Heidelberg, Germany; 4 Geriatric Center Karlsruhe, ViDia Christian Clinics Karlsruhe, Karlsruhe, Germany; 5 Department of Telemedicine, Robert-Bosch-Hospital, Stuttgart, Germany; 6 Institute of Epidemiology and Medical Biometry, Ulm University, Ulm, Germany; 7 Interdisciplinary Centre for Clinical Trials (IZKS) at the University Medical Centre of the Johannes Gutenberg-University Mainz, Mainz, Germany; 8 Medical Faculty, Ruprecht-Karls-University Heidelberg, Heidelberg, Germany

**Keywords:** Geriatric rehabilitation, Post in-house rehabilitation intervention, Patient-related factors, CFIR

## Abstract

**Background:**

Structured aftercare programs are implemented to facilitate the transition from rehabilitation centers to patients’ home environments. Taking the program GeRas as an example, this paper aims to evaluate the influence of patient-related factors on the implementation of the geriatric aftercare program GeRas from patients' and providers' perspectives.

**Methods:**

To capture patients’ and providers’ perspectives, qualitative interviews were conducted using a semi-structured interview guide. The analysis was inductive–deductive and based on the thematic analysis by Braun and Clarke and guided by Domain IV of the CFIR.

**Results:**

16 participants (10 patients, 4 providers, 2 family members) were interviewed from May 2023 to November 2023. Patient-related factors were perceived as an important aspect during the implementation of the GeRas program. The results were allocated to the four Constructs of Domain IV of the CFIR (Motivation, Opportunity, Capability, Needs). Especially patients’ intrinsic motivation, social environment, and physical capabilities seemed to be crucial for successful implementation. While extrinsic motivation can mitigate missing personal capabilities, it cannot replace the presence of intrinsic motivation and capabilities. The results showed that patient-related factors are interlinked.

**Discussion/conclusion:**

The relevance of patient-related factors during the implementation of the GeRas program shows that such programs must consider these factors during intervention planning.

**Supplementary Information:**

The online version contains supplementary material available at 10.1007/s41999-024-01027-5.

## Key summary points

**Aim:**

The aim was to explore how patient-related factors influence the implementation of the geriatric rehabilitation aftercare program GeRas from the perspectives of patients and Healthcare providers.

**Findings:**

Patient-related factors seem to play an important role during the implementation of geriatric rehabilitation aftercare programs. Especially patients’ intrinsic motivation, physical capabilities, and social support were reported to have an effect on successful implementation.

**Message:**

Since the interaction of different patient-related factors seems to play a crucial role during the implementation, they must be considered during planning and implementation of geriatric rehabilitation programs in order to improve effectiveness.

**Supplementary Information:**

The online version contains supplementary material available at 10.1007/s41999-024-01027-5.

## Introduction

German acute and rehabilitation hospitals are obliged to offer discharge management. However, there is usually no systematic handover of patients to community care providers for further case management [1]. To meet the diverse needs of geriatric patients discharged from rehabilitation, a comprehensive aftercare program is needed to address mental, social, as well as physical needs of these patients. Due to the lack of existing agencies within the community, a collaboration of geriatric hospitals with their multi-professional teams and health insurances might overcome this shortcoming. Therefore, the early Qualitative Process Evaluation aimed to evaluate a structured aftercare program, GeRas [**Ge**riatrische **R**eh**a**bilitationserfolge nachhaltig **s**ichern]. GeRas aimed at guiding the transitional process from inpatient rehabilitation to patients’ home environment. The program includes an evidence-based exercise program designed to prevent falls and improve mobility in older patients as well as consultations concerning patients’ nutrition, independence, self-sufficiency, and possible insurance benefits [2, 3].

Due to the diversity within the population of geriatric patients, a variety of factors need to be considered to guarantee a successful implementation of aftercare programs [4]. Patient-related factors play a particularly important role in the design of exercise programs for older patients but may be overlooked [5, 6]. Boulton and Horne found, that individual factors, such as people’s ability, motivation, beliefs, and personality as well as social and environmental factors, affect older people's participation in physical activity and often override extrinsic factors which may be easier to influence by policymakers [7]. Although fall prevention programs can be considered a type of physical activity, they impose specific implementation requirements due to their often multimorbid target population. Research has shown that some patients participate in fall prevention programs while others will not participate in the same program. Therefore, further research is necessary to identify factors associated with patient's willingness to engage in these programs [8]. Fernandes et al. suggest that healthcare system gaps, social context, economic context, health status, psychological capability, as well as lack of knowledge may be associated with older people's adherence to fall prevention programs [9]. Previous research has confirmed that patients' characteristics are a relevant factor during the implementation of aftercare programs [10, 11]. Continuous participation in the program is essential for achieving effects. Understanding and addressing the reasons for non-adherence is important for treatment outcomes.

In order to successfully implement any intervention, including exercise programs, several factors and determinants, including individual characteristics and patient-related factors, must be considered [12]. Many frameworks have been developed to simplify and structure these factors, one of the most frequently used being the Consolidated Framework for Implementation Research (CFIR) [13]. Since patient-related factors seem to play an important role in the implementation of rehabilitation programs, this paper aims to focus on the role of these factors. The domain ‘individual factors’ of the CFIR is divided into two subdomains and defines the roles and characteristics of individuals involved in the implementation of an intervention. Several studies have explored the role of these factors in implementing exercise programs as well as programs with telehealth components [14, 15]. However, research evaluating these factors in rehabilitation aftercare programs for older patients is scarce. Especially research focusing on patients' perspectives concerning patient-related factors associated with the implementation of aftercare programs. Thus, this paper aims to answer the following research question: *How do patient-related factors influence the implementation of the geriatric rehabilitation aftercare program GeRas from the perspectives of patients and Healthcare providers?*

## Methods

The early Qualitative Process Evaluation discussed in this paper is based on the GeRas study. The GeRas study is a three-center, assessor-blinded, randomized, controlled, parallel-group trial consisting of a three-month multimodal home-based intervention program following discharge from inpatient geriatric rehabilitation centers [2]. The intervention is delivered either by home visits and telephone calls (conventional intervention) or using a combination of tablet computers in an eHealth system as well as home visits (tablet intervention). A detailed description of the project can be found in the published study protocol [2].

### Study design

For this early Qualitative Process Evaluation of the implementation of the GeRas program, semi-structured qualitative interviews were conducted. To ensure that different perspectives on patient-related factors, which may play a role in the implementation process, are considered patients and healthcare providers (HCP) involved in the project were interviewed.

### Study setting (GeRas)

The intervention was implemented and evaluated at three different study sites (1) Robert-Bosch-Hospital Stuttgart, Germany (2) Agaplesion Bethanien Hospital Heidelberg, Germany (3) ViDia Christian Clinics Karlsruhe, Germany [2].

### Study population of the early qualitative process evaluation

#### Patients

All patients who were included in the main study between October 2022 (start of the main study) and October 2023 and participated in the intervention groups, receiving the conventional or tablet intervention, were invited to take part in an interview following the three-month intervention period. To participate in the Qualitative Process Evaluation, patients had to have completed the three-month intervention period of the GeRas program, be fluent in German, and give their written informed consent.

#### Healthcare providers

Physical therapists employed at one of the participating geriatric rehabilitation centers were invited to participate in an interview. The following inclusion criteria had to be fulfilled: involvement in the implementation and delivery of the conventional or tablet intervention in one of the three study sites, fluency in German, older than 18 years, and written informed consent to participation in an interview.

### Intervention (GeRas)

The patients of both intervention groups receive a multimodal home-based intervention program to improve mobility and social participation (GeRas program) [2]. The GeRas intervention starts immediately upon discharge from geriatric rehabilitation and lasts three months. It is delivered by a multidisciplinary team of social workers, physical therapists, and geriatricians employed at the discharging rehabilitation center as well as social workers employed at patients’ health insurance, the AOK Baden-Württemberg. The GeRas program consists of the following components: (a) an outpatient physical exercise program, (b) outpatient care counseling, (c) assessment of person-environment fit, and (d) nutrition advice [2]. A detailed description of the intervention can be found in the published study protocol [2].

### Implementation activities

Several implementation activities were conducted to promote successful implementation. In terms of the ERIC Taxonomy [16], most strategies concerned educational meetings. Further information can be found in the supplementary material.

### Sampling and recruitment

#### Patients

All patients who were assigned to one of the intervention groups received an informational letter and an informed consent form after they had completed the three-month intervention period by post, which they had to send it back to the research team that was responsible for the Qualitative Process Evaluation at the Department of General Practice and Health Services Research of the University Hospital Heidelberg in case they were interested in participating in the Qualitative Process Evaluation. Patients were then contacted by a member of the research team responsible for the Qualitative Process Evaluation via telephone to arrange an appointment.

#### Healthcare providers

To recruit HCPs, the research team responsible for the Qualitative Process Evaluation provided envelopes that included an invitation letter to participate in the Qualitative Process Evaluation, the information leaflet, and an informed consent form for audio-recording the interviews. The internal project coordinator at each study site was responsible for the distribution of the envelopes. HCPs who decided to participate were requested to contact the research team directly via e-mail or phone.

Additional strategies, such as e-mail reminders and team meetings, were utilized to increase the response rate for both, patients and HCPs.

#### Interview guide

The interview questions explored patients' and HCPs' experience with the aftercare program GeRas and were developed based on the CFIR and the RE-AIM Frameworks [13, 17]. The interview guide was developed and discussed within a qualitative research colloquium at the Department of General Practice and Health Services Research in Heidelberg. All interview questions were open-ended and focused on the perceptions and experiences of patients and HCPs involved in the GeRas study.

### Data collection

All patients were interviewed by a female researcher with a background in health services research and occupational therapy between June 2023 and November 2023 [LM]. HCPs were interviewed by a female researcher with a background in health services research and nursing between May 2023 and November 2023 [CR]. Interviews with patients were conducted after they had completed the three-month intervention period. The implementation of the intervention started in October 2022, the first patient completed the three-month intervention period in January 2023, and in June 2023 the first patient consented to participate. Thus, the period of the interviews (May 2023–November 2023) captures early perspectives and experiences. For two of the patient interviews, a family member was present upon request of the patient. The patient interviews were conducted either via telephone or in person at the University of Heidelberg or patients’ homes according to participants' preferences. Provider interviews were conducted via telephone.

Interviews were digitally audio-recorded. No additional field notes were collected and none of the interviews was repeated. One patient interview was not digitally recorded due to the withdrawal of consent. Notes were taken by the interviewer during the interview and later written down in the form of a verbatim record. It was coded in the same way as the remaining transcripts.

The interviews were pseudonymized and transcribed using the software f4transkript. After transcription, interviews were reviewed while listening to the audio records to ensure accuracy. No transcripts were returned to participants for correction. Data is kept in a secure place according to data protection guidelines at the Department of General Practice and Health Services Research.

### Data analysis

Using an inductive-deductive approach, the qualitative analysis was guided by the Consolidated Framework for Implementation Research (CFIR), specifically by Domain IV “Individuals Domain” within which the characteristics subdomain was utilized to derive four deductive themes [13]. This Domain describes person-related factors and characteristics and therefore caters to the answering of the research question. The Individuals Domain of the CFIR uses three constructs of the COM-B behavioral change model, “Capability,” “Opportunity” and “Motivation”, the construct “Needs” was added by the authors of the CFIR as an additional construct [13]. Previous research suggests using frameworks as guidance while keeping an open research approach and developing inductive codes and themes within research data to not lose important information [18]. Therefore, deductive and inductive approaches were combined during the analysis.

To increase intersubjective comprehensibility, all interviews were analyzed separately by two female researchers [LM and CR]; codes were subsequently discussed to unify any differing codes. Codes were then grouped into themes according to the steps of reflexive thematic analysis by Braun and Clarke, themes were then allocated to the constructs of the CFIR (Domain IV “Characteristics of Individuals”) [19]. The analysis software MAXQDA version 2022, a computer-assisted qualitative data management software was used to structure and assist with the analysis. All quotations used in this paper were translated into English, some were adapted to maintain meaning.

### Ethical approval

The study was approved by the local ethics committees at each study site (Heidelberg: Ethics Committee of the Medical Faculty of Heidelberg University [approval # S-395/2022]; Stuttgart & Karlsruhe: Ethics Committee of the State Medical Association Baden-Württemberg [approval # B-F-2022-057]). Written informed consent for audio recording the interviews was provided prior to the interview by participants to ensure adequate opportunity for consideration. The study was reported guided by the Consolidated Criteria for Reporting Qualitative Studies (COREQ) checklist for qualitative research [20].

## Results

### Overview

A total of *n* = 16 participants were interviewed over the course of seven months (May 2023–November 2023). Of these, 10 interviews were conducted with patients, while four were conducted with HCPs. Family members participated in two patient interviews upon request of the patients. The participation rate was 17.9% for patients (10 out of 56 patients who were initially invited and had completed the three-month intervention period (between October 2022 and October 2023) and 80% for HCPs (4 out of 5 HCP who were initially invited participated in an interview). The participants' characteristics are displayed in the following table (Table 1).

**Table 1:** Characteristics of participants

Participants	Healthcare providers *N* = 4	Patients *N* = 10	Family members *N* = 2
Age range (median) in years	40.5 Min 28 Max 52	81 Min 75 Max 91	Not applicable*
Gender	Female: 4 Male: 0	Female: 6 Male: 4	Female: 2 Male: 0
Years of experience Median (range)	13 (5–21)	Not applicable	Not applicable
Intervention group (patients)	Not applicable	Poster: 7 Tablet: 3	Poster: 2 Tablet: 0
Location	Heidelberg:1 Karlsruhe:1 Stuttgart:2	Heidelberg:3 Karlsruhe:3 Stuttgart:4	Heidelberg: 0 Karlsruhe: 1 Stuttgart: 1
Format	In-person: 0 Phone: 4	In-person: 7 Phone: 3	In-person: 2 Phone: 0
Interview length in minutes, median (range)	31.18 Min 22.5 Max 40	37.5 Min 22 Max 54	

### Results of the qualitative analysis

**Fig. 1:**
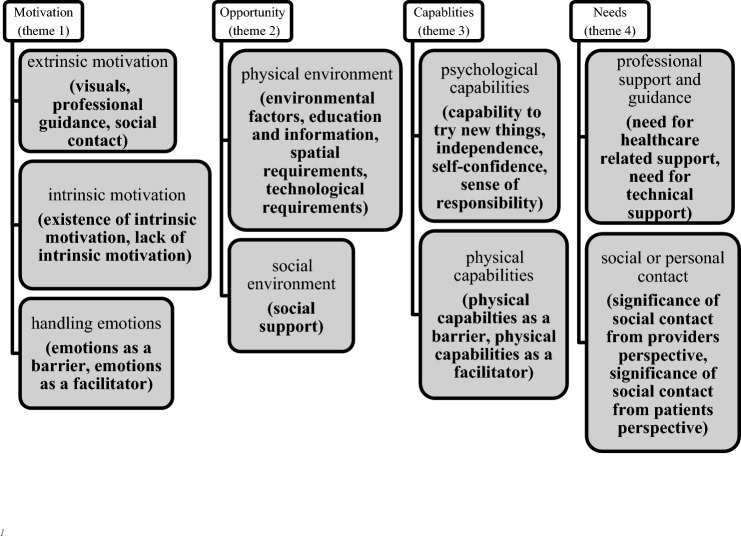
Main themes and subthemes that emerged from the analysis (Fig. 1 shows the four deductively derived main themes (white boxes); gray boxes show subthemes within the four main themes and inductively created codes within brackets)

The interviews showed a wide range of statements and themes related to the four main constructs of the CFIR Domain IV (“Individuals Characteristics”). Subthemes that resulted from the analysis were allocated to the four main themes deductively provided by the CFIR Domain IV “Characteristics of Individuals” constructs (“Motivation”, “Opportunity”, “Capability” and “Needs”). The themes within Opportunity and Capabilities were guided by the COM-B model, while themes within Motivation and Needs were formed solely inductive. The four themes as well as the allocated subthemes are portrayed in the following figure (Fig. 1).

### Influence of “Motivation” (theme 1)

**Fig. 2:**
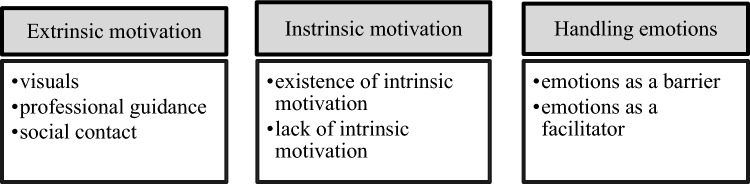
Subthemes within the main theme “Motivation”

Figure 2,^1^ shows the subthemes and inductive codes allocated to theme 1: motivation. Results regarding this theme are presented below.

### Extrinsic motivation

Extrinsic motivation provided by the program was evaluated as a facilitator to successful participation by all participants. From the patients' perspective, a lack of extrinsic motivation may lead to negligence. As the program progressed over time, some patients found it difficult to stay motivated if extrinsic motivation was no longer given. Patients also advocated that greater motoric disabilities indicated a higher need for extrinsic motivation.

“Extrinsic motivation plays a bigger role when you are less fit. The program would need to be designed differently for these people, maybe you could build in more home visits to enhance extrinsic motivation and build up a certain habit. The tablet does not give enough motivation.” (PAT1, Pos. 36)^2^

Three main sources of extrinsic motivation were reported by participants: *visuals, social contact, and professional guidance*. Patients stated that having the exercises depicted on the poster helped them stay motivated and reminded them to exercise daily. Despite the poster being more present in the patient's home environment, some patients reported the same effect through the videos on the tablet computer. Providers felt this age group specifically benefited from the visual depiction of exercises via the poster as it facilitated patients’ motivation and promoted a sense of security. They also emphasized the importance of choosing a prominent location for the poster, as it acted as a reminder and can therefore influence patients’ participation in the program.

“Well, what plays a big role within this age group I think, are the visuals and especially, that they could touch the poster, actually look at the pictures directly, that they were not distracted by technology.” (TR1, Pos. 14)^3^

Patients described that the social contact initiated by the HCP team through home visits and phone calls had a positive impact on their motivation. Some stated that the social contact had a higher impact on them than the exercise program itself.

“As I said, to me, it was positive to see progress and that someone looks after me. Through the phone calls and that someone came by again and again and talked to me […] those are aspects that I actually simply perceived as positive.” (PAT5, Pos. 94)

This impression was supported by provider statements. They consistently reported that social contact played an important role in motivating patients to continue the program. Providers additionally emphasized that continuous weekly contact helped keep patients motivated.

Professional guidance given by the HCP team was also perceived as an extrinsic motivation. Patients felt they could rely on the instructions given by the team which in return made them feel secure and taken care of. One patient stated that professional guidance in person was of higher relevance for patients with greater motor disabilities. Providers observed that their instructions played an important role when it came to motivating patients. According to providers, motivation could be improved by talking to patients about their worries and fears but also by creating a feeling of control.

### Intrinsic motivation

Intrinsic motivation was described as the basic requirement for successful participation in the program, especially due to the high demand for autonomous exercising which is part of the program. According to patients, their intrinsic motivation was driven by the goal of improving mobility, independence, and quality of life. In addition, patients who talked about having a general interest in their health and were socially integrated showed higher intrinsic motivation to participate in the program.

“And then it is people of course, that are willing to do something. Most of the time people who still have lots of social contacts […] that still actively organize their free time, they want to and they participate quite well because they actually have a goal in mind.” (TR2, Pos. 56)

A high level of intrinsic motivation also helped patients reach sustainable improvements even after the intervention period was completed. Due to the reduced amount of extrinsic motivation provided, participants of the tablet intervention group felt that a higher level of intrinsic motivation was required in comparison to the conventional intervention. All patients agreed that a lack of intrinsic motivation cannot be fully compensated by extrinsic motivators. Providers also stated they found it difficult to motivate patients who lack intrinsic motivation. Simultaneously, they talked about having to respect patients' lack of motivation and expressed that it often correlated with little remaining life goals and old age.

“If people participate in the program and don’t do anything after that, only sit in front of the TV or likewise, then it doesn’t work. Then everything you have achieved is lost.” (PAT2, Pos. 100)

Without intrinsic motivation, patients may not be able to benefit from the aftercare program according to HCPs. They reported that to achieve improvements, patients needed to be intrinsically motivated to participate in the program. Patients also described that the program may not be suited for patients who lack intrinsic motivation or have no interest in improving their mobility. Lacking intrinsic motivation was perceived as the main barrier regarding the participation goals and continuous compliance of the patients, which are necessary to achieve sustainability of the intervention program.

“Well, my impression was, that it was, alright, but through my own negligence, when no one was there, I often forgot, and so on. So, if someone would have been behind it more often it would have been more intense.” (PAT4, Pos. 2)

### Handling emotions

Different emotions were talked about by patients and HCPs. The most prominent emotion seemed to be frustration which was caused by technical difficulties. Patients described that frustration became a main barrier to their motivation. Frustration caused by technical challenges reduced patients motivation and patience with the program itself. In addition, some patients mentioned being disappointed by the program due to consistent technical difficulties. Overall, emotions talked about in the tablet group seemed to be more negative compared to emotions described by the poster group. Providers described a vicious cycle developing from technical difficulties in combination with feelings of frustration which may lead to a decreased motivation for the continuation of the program.

“It really is the frustration that increases with increasing technical challenges and, the motivation to perform the exercises automatically decreases and then we have a vicious circle, right.” (TR1, Pos. 19–20)

Although the tablet seemed to cause mostly negative emotions, HCPs also experienced some positive emotions in patients. Positive emotions such as joy or pride were observed once patients managed to handle the tablet computer, leading to an improvement in their self-confidence, according to HCPs.

### Influence of “Opportunity” (theme 2)

**Fig. 3:**
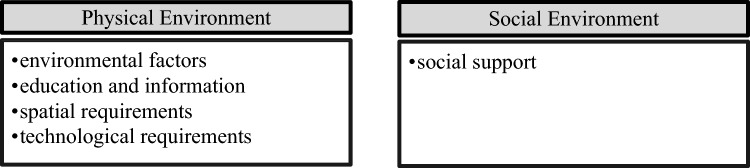
Subthemes within the main theme “Opportunity”

Figure 3,^4^ shows the subthemes and inductive codes allocated to theme 2: Opportunity. Results regarding this theme are presented below.

### Physical environment

A range of factors within the physical environment were described by patients and HCPs. The main subthemes that emerged were *environmental factors, education and information, spatial requirements,* and *technological requirements*. Weather conditions such as extreme heat or rain influenced patients’ ability to complete exercises (daily walk). Patients and providers reported that some patients’ homes did not meet the spatial requirements to execute exercises as planned. Limited space and non-existent stairs which were needed to perform the strength exercises within the exercise program seemed to be the most common barrier and restricted patients from performing exercises.

“We do not have a sports room in our assisted living establishment, the common room cannot be used either, which leaves only the corridor, without handholds, you have to touch along the wall, it’s not easy.” (PAT6, Pos. 74)

Additionally, living in rural areas was described as a facilitator of participation because patients found it easier to perform their daily walk within surroundings close to nature.

The aspect of education was only talked about by patients. They expressed that to increase the opportunity to participate, patients should be educated and informed extensively concerning the procedure and possible benefits of the program. Technological requirements were described as especially important for patients working with the tablet. Patients and providers stated that an unstable Wi-Fi connection at home and a weak mobile internet connection were the most common problems hindering the tablet intervention.

### Social environment

The subtheme *Social support* was described as an important requirement for participation. Patients who had family members or partners who supported them were grateful and stated that their support contributed to the opportunity to participate in the program.

Providers additionally observed that patients living with partners or family members had fewer difficulties and were more likely to benefit from the program. Therefore, family members and partners were regularly involved in the program. Especially patients with greater motoric disabilities seemed to profit from social support.

“it makes a big difference and that is what I meant by the different settings, that has such a big impact, whether family members are still available or whether someone is on their own, both cases and it’s quite fascinating how that has an impact.” (TR1, Pos. 48)

### Influence of “Capability” (theme 3)

**Fig. 4:**
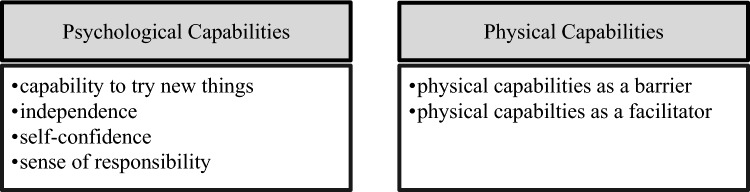
Subthemes within the main theme “Capabilities”

Figure 4,^5^ shows the subthemes and inductive codes allocated to theme 3: capability. Results regarding this theme are presented below.

### Psychological capabilities

Psychological capabilities, such as “capability to try new things”, “independence”, “knowledge”, “self-confidence”, and “sense of responsibility”, seemed to have an impact on the participation in the program. Especially patients assigned to the tablet group frequently addressed perseverance and knowledge in terms of digital competencies. Patients felt that their lack of technological knowledge prohibited their ability to work with the tablet computer. HCPs also described a generational difficulty as they felt geriatric patients did not have adequate knowledge of the technology required to work with the tablet computer effectively. This is described as one of the main barriers to implementation. Some providers suggested waiting 5–10 years to reach a knowledgeable, probably more tech-savvy, generation.

“I know from a lot of people, that they just can’t handle smartphones very well, and that is how I feel as well […] it will be different in years to come because people just grew up differently and get into it differently than us old people, where that simply did not exist.” (PAT8, Pos. 88–91)

This is highlighted by a patient who described that she was opposed to working with the tablet from the very beginning. This had a high impact on her participation in the program. This reaction was also observed by providers. They reported that some patients simply did not want to work with the tablet and were relieved to have been assigned to the poster group because they did not want to be confronted with unfamiliar technology.

Some patients showed a high level of perseverance which seemed to promote their ability to overcome difficulties and enabled them to continue the program even when motivation decreased. High levels of perseverance also seemed to alleviate patients' lack of knowledge. Providers expressed that perseverance was particularly relevant due to the long intervention phase including autonomous, daily exercise, and technical difficulties within the tablet group.

“Yes, I did them by myself, the exercises. I didn’t miss any exercises even when the tablet did not work. Now more than ever.” (PAT2, Pos. 26)

Several patients described that their will to regain independence was the primary goal for their participation in the program. Patients who led an independent lifestyle before their hospitalization reported benefitting from their need for independence and independently performing exercises outside of the program. Patients who were in the tablet group reported they had to be particularly independent due to ongoing technical difficulties. Self-confidence was described as both a requirement for and a result of participation in the program. Patients' self-confidence was especially promoted by noticeable, early improvements.

Psychological factors, more specifically good mental health was described by patients as a requirement for participation. They found it important to overcome psychological barriers before participating in the program as they associated psychological disabilities with lower levels of intrinsic motivation and self-confidence. Providers perceived cognitive abilities to be a common psychological barrier, mental health was not mentioned.

“Of course, if you are depressed, I could imagine, if you say, it’s not going to help anyways, that those people profit less from the program.” (PAT9, Pos. 54)

### Physical capabilities

Physical capabilities were discussed both as a *barrier* and a potential *facilitator*. Patients who reported fewer physical disabilities also reported fewer problems regarding the execution of exercises. HCPs confirmed this, observing that physical capabilities were a crucial factor for successful participation. Although the program can be adjusted by the physical therapists, a lack of physical capabilities was perceived as a barrier to participation especially in the initial intervention phase directly after discharge. A lack of strength, impaired vision, sensory disorders, severe pain, and pre-existing conditions seemed to have a high impact on the ability to correctly perform the exercises within the program. Patients reported that exercises took longer or had to be interrupted due to physical disabilities, which caused frustration. These difficulties seemed to decrease as the program progressed and patients gained motoric capabilities, such as strength and balance.

“The exercises were a bit too much for me sometimes. At least at the beginning, when I was told, this ten times and that ten times, that was a bit too much for me at the beginning with my weakness.” (PAT5, Pos. 60)

Providers reported that physical disabilities such as injuries, edemas, or high pain levels hampered the implementation of the tablet intervention because physical therapists were not able to physically see and control movements which limited their abilities to respond appropriately. They also stated that some patients were only able to perform the exercises on the lowest level of difficulty due to their restricted physical capabilities. This limited their ability to experience improvements during the program.

“The strength exercises, you need more time due to the breathing, because we had to take breaks again and again. So, the real problem was actually the breathing, less so the strength. And the endurance respectively. Due to the breathing, you cannot do much. At some point, you always reach the limit.” (PAT9, Pos. 28)

### Influence of patient “Needs” (theme 4)

**Fig. 5:**
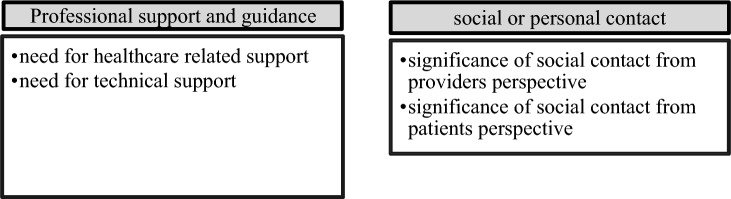
Subthemes within the main theme “Needs”

Figure 5,^6^ shows the subthemes and inductive codes allocated to theme 4: Needs. Results regarding this theme are presented below.

### Professional support and guidance

The most frequently addressed need is the *need for healthcare-related support*. Statements by patients showed a high need for healthcare-related support by physical therapists and social workers. Professional support was experienced as helpful for the explanation and correction of exercises, to give advice, and also functioned as a reminder. Patients described particularly benefitting from the initiative taken by HCPs.

Providers also observed a high need for healthcare-related support specifically during the initial transition from the rehabilitation hospital to the patients’ home environment.

“I found that great, that’s why I signed it right away because I thought, then I will have something at home and don’t have to directly go into town, because I would not have been able to walk and would have constantly needed someone to drive me.” (PAT8, Pos. 3–4)

Additionally, the *need for technical support* was addressed by one patient who stated that she required a high amount of technical support to participate in the program and work with the tablet computer. Providers described the need for technical support more frequently and stated they had to spend more time than expected to meet patients' need for technical support.

### Social or personal contact

The *significance of social contact* was addressed by providers and patients alike. Providers emphasized that a stable therapeutic relationship was particularly important when working with geriatric patients. They stated that an initial personal contact was of high importance, particularly in this study population. Although one provider stated that she was able to maintain a better relationship with patients through video calls, home visits cannot be adequately replaced according to most providers.

“I find that the digital contact simply can’t replace the personal contact adequately but it can of course be, so I find, used well additionally.” (TR2, Pos. 60)

“That is very important, isolation prevention. Social competence, sociability, simply letting patients speak from their soul.” (TR4, Pos. 74)

Patients described that social contact was needed to feel cared for and build a stable relationship with the HCP. Some patients additionally emphasized that the quality and personal fit of the patient-provider relationship as particularly relevant. Some patients described feeling lonely and socially isolated which increased their need for social contact. Patients additionally described that social contact with the study personnel gave them a feeling of security. As described in 3.1.1, social contact was oftentimes mentioned as one of the main motivations to participate in the GeRas program.

“If I had been alone, ghosts would have appeared. And to always have the feeling, you can call someone. And to know, next week they’re going to visit again. That was, that was positive.” (PAT3, Pos. 42)

## Discussion

This paper aimed to answer the following research question: *How do patient-related factors influence the implementation of the geriatric rehabilitation aftercare program GeRas?* The findings demonstrate that patient-related factors play an important role during the implementation of rehabilitation programs in geriatrics, confirming that the personal, psychological, and social characteristics of patients are relevant and should be considered during the implementation of aftercare programs in this population [11].

All participants, patients as well as HCPs, discussed patient-related factors during the interview, without being explicitly asked to do so. The interview data showed a thematic saturation as new interviews did not provide additional information. Thematic saturation was discussed within the research team during the analysis process and was reached when no new themes were derived from additional interviews. This underlines the importance of patient-related factors. While all four constructs within the CFIR Domain “Individuals Characteristics” were addressed by all participants, motivation (theme 1) and capability (theme 3) were discussed most frequently. The results showed that intrinsic motivation and perseverance were crucial for participation in the program and a lack therefore cannot be fully compensated by extrinsic motivators.

Participants who were physically disabled encountered more difficulties with the exercises, especially during the initial intervention stage directly after discharge. The interviews showed that frustration and rejection of new technologies acted as barriers during the implementation of the tablet intervention. This seemed to be amplified by a lack of technical knowledge within the population of geriatric patients. Generally, the analysis of the interview data revealed that patient factors should not be addressed individually but rather as a mutually dependent system of interlinked factors.

The role of ***motivation**** (theme 1)*, especially intrinsic motivation was perceived as a main factor influencing patients’ ability to participate in the rehabilitation program. This supports the findings of earlier studies declaring patients' motivation as the main driving factor for successful participation in rehabilitation programs [10, 11, 21, 22]. Lack of intrinsic motivation was described as the main barrier to participation. HCPs and patients perceived it to be a pre-existent requirement for the rehabilitation program. Both groups argued that participants who lack intrinsic motivation were hard to motivate, especially because the program requires patients to train autonomously. HCPs could, however, partly compensate for a lack of intrinsic motivation by providing extrinsic motivation, highlighting the interaction of intrinsic and extrinsic motivation as an important influencing factor [10]. Patient-centered goals and finding meaning behind the exercises seemed to additionally increase patients’ intrinsic motivation and therefore facilitate sustainable improvements. Setting specific, patient-centered goals may additionally increase adherence to new technologies [23].

Within the GeRas intervention, the most motivating extrinsic factors were perceived to be the social contact provided by the HCP team, professional guidance by physical therapists and social workers, and visual depiction of exercises displayed either by the poster or by videos. Beyond a positive effect on motivation, visual depiction and personal guidance by physical therapists have been proven to improve exercise execution in older adults [24]. The outreach character (e.g., planned telephone call by social workers to discuss additional service needs) of the intervention was perceived as especially motivating by patients and HCPs equally. Patients who lacked intrinsic motivation at the beginning of the program seemed to gain confidence and motivation through these extrinsic factors.

Intrinsic motivation seemed to be related to a high level of independence, self-confidence, and perseverance. Highlighting the relevance of patients’ psychological ***capabilities**** (theme 3)*. Perseverance seemed to be especially important for patients in the telemedicine group. Due to technical difficulties at the beginning of the implementation period, the tablet did not always function as planned leading to high levels of frustration. As previous research suggests, negative emotions such as frustration can be a barrier to physical activity [21, 22].

Patients’ lack of digital competencies seemed to further contribute to the frustration, highlighting the demand for further education of older patients and the generation-specific difficulties in the usage of new technologies. Concerning adherence to telehealth interventions, previous research has also found a lack of knowledge to be a main barrier [15]. Although a lack of digital competencies was reported by most participants, HCPs should constantly reflect on their own beliefs to counteract the development of prejudices concerning older persons’ digital competencies [25]. Another frequent issue concerning patients’ digital competencies knowledge seemed to be insufficient education before the aftercare program. This is especially crucial for patients' motivation and understanding of the program [10, 26].

As previous studies have shown, a lack of physical ***capabilities**** (theme 3)* seemed to be one of the greatest barriers to participating in exercise programs for geriatric patients [9]. Patients and HCPs reported impaired vision, muscular weakness, edemas, and injuries to be the most common physical barriers. Participants' statements additionally highlighted that limited physical capabilities inhibit the opportunity to experience success and improvements. Therefore, intrinsic and extrinsic motivation seemed to be especially relevant for those with low physical capabilities to continue participation despite a lack of subjective improvement [10]. As former studies have shown, the lack of physical disabilities seems to lead to decreased intrinsic motivation and self-confidence because patients realize they are not able to perform exercises as planned [27]. Especially during the early intervention stages, it is crucial to experience success and improvements to facilitate long-term motivation [10]. This can be achieved through extrinsic motivation provided by HCPs. Patients with greater disabilities therefore had a higher need for professional guidance and individual treatment options.

The ***opportunity**** (theme 2)* to participate seemed to be more frequently influenced by the social environment compared to the physical environment. Patients living with a partner or receiving social support through family members seemed to have fewer problems executing the intervention. Social support seems to mitigate the hindering effect of physical disabilities and cognitive restrictions according to statements by HCPs and patients. This supports previous research that highlights social context as a main barrier to undertaking prevention programs in geriatrics [9]. The physical environment, such as special requirements or environmental factors, seemed to have a minor effect on the implementation.

The interview data showed geriatric patients' high ***need**** (theme 4)* for social contact and medical support which are not met by current outpatient care. According to previous research, patients' needs should be considered during intervention planning as this may enhance patients' motivation [10]. Patients' needs for social contact and support were met by the GeRas intervention as patients report social contact through the study team to be an extrinsically motivating factor. Particularly through the provision of initial and ongoing home visits, depending on the intervention group, as well as weekly telephone calls, the GeRas intervention seemed to attend to these needs.

Positive social interactions with study personnel have been found to potentially increase adherence [28–30]. This is even more relevant during the transition between healthcare sectors [31]. Participants of GeRas backed these findings, stating that provider and patient relationships played an important role. Especially since providers acted as extrinsic motivators and therefore facilitate successful participation. Since social contact has also been shown to improve self-confidence and optimism, it seems to be an important factor for successful implementation and should therefore be promoted [30].

The results of this Qualitative Process Evaluation showed that aspects related to patients' characteristics, such as motivation, physical capabilities, and confidence, were crucial for the successful implementation of the GeRas aftercare program and were closely linked to each other. The interrelation between different patient characteristics should therefore be further investigated.

### Limitations

Some limitations of the study must be acknowledged. The study was conducted using a small sample of patients and HCPs in southern Germany working in three hospitals. Thus, results may differ within a different study population and geographic region. Although thematic saturation was reached, more patients randomized to the poster intervention than to the tablet intervention were interviewed which may have impacted the results. More interviews with patients randomized to the tablet intervention may have led to more diverse results. It must also be taken into consideration that patients and HCPs who agreed to be interviewed may have different perceptions and experiences compared to those who did not participate in an interview. Especially with regard to patients’ intrinsic motivation, it can be assumed that patients willing to participate in an interview were generally more motivated and therefore have a positive attitude toward exercising. Answers may have been skewed by social desirability although interviewers did not perceive this to be a problem during the interviews. Quotes used within the study were translated from German into English. Therefore, it is possible that the meaning transported in translated quotes may differ slightly from the original meaning in German.

## Conclusion

Patient-related factors seemed to play an important role in the successful participation in the GeRas aftercare program. Especially factors related to patient’s intrinsic motivation (theme 1) and physical capabilities (theme 3) can act as crucial barriers or facilitators to successful implementation and should therefore be considered during implementation planning. Patients’ social environment (theme 2) can influence their opportunity to participate in the program through support and motivation provided by family members or friends. Additionally, the interview data showed that patient-related factors seem to be closely intertwined. Especially the aspect of intrinsic motivation seems to be strongly influenced by factors related to patients' psychological and physical capabilities. For example, a high level of intrinsic motivation seemed to be associated with increased perseverance. A low level of physical capabilities on the other hand seemed to increase frustration and reduce intrinsic motivation.

Regarding the eHealth intervention, patients' lack of digital competencies (theme 3) seemed to be the main hindering factor. The conventional intervention seemed to be slightly more accessible compared to the tablet-based intervention, due to fewer requirements and competencies needed. In addition, patients' needs (theme 4) seemed to be largely unmet in the usual-care situation currently. The GeRas program seems to overcome this issue by continuing social contact after discharge to the home environment. As the consideration of patient needs leads to higher compliance to aftercare programs, future research should focus on expanding knowledge regarding geriatric patients' needs after rehabilitation.

## Supplementary Information

Below is the link to the electronic supplementary material.

Supplementary file1 (DOCX 14 KB)

## Data Availability

The dataset that was generated and analyzed during the study will not be made publicly available due to European and German data protection law.

## Footnotes

1 Figure 2 shows subthemes within theme 1 (gray boxes) as well as codes that emerged inductively (white boxes).

2 Quotations by Patients are marked “PATx”, “PosX” refers to the position of the quotation within the transcript.

3 Quotations by HCPs are marked “TRx”, “PosX” refers to the position of the quotation within the transcript.

4 Figure 3 shows subthemes within theme 2 (gray boxes) as well as codes that emerged inductively (white boxes).

5 Figure 4 shows subthemes within theme 3 (gray boxes) as well as codes that emerged inductively (white boxes).

6 Figure 5 shows subthemes within theme 4 (gray boxes) as well as codes that emerged inductively (white boxes).
