# Supplementary material for: The role of patient-related factors in the implementation of a multimodal home-based rehabilitation intervention after discharge from inpatient geriatric rehabilitation (GeRas): a qualitative process evaluation
Source: Eur Geriatr Med. 2024 Jul 31;15(5):1369–82. doi: 10.1007/s41999-024-01027-5 (PMC11614932; doi:10.1007/s41999-024-01027-5)
Supplement: Supplementary file 1 — Supplementary file1 (DOCX 14 KB) [file 41999_2024_1027_MOESM1_ESM.docx]

| ERIC strategy | implementation activities | Format | Participants | Content | Date |
| --- | --- | --- | --- | --- | --- |
| Conduct educational Meetings | Trainer training | In person | Trainers  Clinical study coordination | Procedure of the study  Implementation of the training  Trainer tasks | September 2022 |
| Conduct educational meetings | Social service training | In person | Clinic social service  Insurance social service  Clinical study coordination  project evaluation | Coordination of the interface between clinic and insurance social service | September 2022 |
| conduct educational meetings | Clinician training | Online | Clinicians | Procedure of the study  Clinician tasks | September 2022 |
| conduct educational meetings | Software training | Online | Trainers  Clinical study coordination  Clinical study monitoring  Project evaluation  Clinical study doctors  Clinic social service  Insurance social service  University Ulm | Handling of the software | Part 1: August 2022  Part 2: October 2022 |
| conduct educational meetings | Tablet training | In person | Trainers  Clinical study coordination  Clinic social service  Insurance social service | Handling of the tablets | September 2022 |
| involve patients/consumers and family members | Patient advisory board | In person | Representatives of patients  Researchers (clinical study coordination; clinical study doctors; project evaluation) | Part 1: Presentation of the project; Discussion of study material  Part 2: Presentation of results of the first meeting; Testing of the training app and tablets  Part 3: Optimization of Recruitment; Acceptance of tablets  Part 4: to be determined | Part 1: April 2022  Part 2: July 2022  Part 3: May 2023  Part 4: 3. Quarter 2024 |
| organize clinician implementation team meetings | Trainer workshop | Online | Trainers  Clinical study coordination | Discussion of current changes and/ or problems; collegial exchange; ascertain standard procedure | 1 meeting per quarter (continuously) |
